# Turnover of professional nurses at Mokopane Hospital in the Limpopo Province, South Africa: Experiences of nursing unit managers

**DOI:** 10.4102/curationis.v38i2.1566

**Published:** 2015-12-17

**Authors:** Mogale L. Mmamma, Tebogo M. Mothiba, Malema R. Nancy

**Affiliations:** 1 Department of Nursing Science, University of Limpopo, South Africa

## Abstract

**Background:**

Staff turnover of professional nurses remains a concern for public and private hospitals management because it has an impact on the morale of nurses and it may also lead to poor patient care.

**Objectives:**

The objectives of this study were to explore and describe the experiences of nursing unit managers with regard to the turnover of professional nurses who were under their supervision.

**Method:**

A qualitative, explorative, descriptive research design was used to determine the experiences of nursing unit managers related to the turnover of professional nurses. Data collection was done by using semi-structured one-to-one interviews with professional nurses. Two groups of participants were interviewed: Those working day duty (*n* = 9) and those working night duty (*n* = 3) who were at work on the anticipated days for data collection.

**Results:**

The findings revealed that every unit was experiencing a shortage of professional nurses, which caused other nurses to work overtime with an inevitable increase in workload. That led to tiredness, conflict amongst professional nurses, job dissatisfaction, and absenteeism which compromised nursing care. This resulted in patient dissatisfaction and sometimes led to deaths that could have been prevented.

**Conclusion:**

It is recommended that staff turnover should be addressed by the hospital top management implementing several strategies. For example, top management could ensure that staff members work in a healthy environment with resources that they need during the provision of care, address the effects of the staff turnover, support the staff members and refrain from putting pressure on nursing unit managers whilst they are attending to problems.

## Introduction and background

Staff turnover is a global problem that annually has dire negative financial implications for organisations because it perpetuates the need to recruit other staff members to replace the ones who have left and also to remunerate the ones who are obliged to work overtime. It also causes job 1 dissatisfaction, stress and burnout amongst committed members of staff who wish to remain at the affected organisations (Selebi & Minnaar 2009:31). Mudor and Tooksoon (2011:41) indicate that job satisfaction plays an important role in staff turnover, whilst staff turnover is regarded as the voluntarily or involuntarily movement of employees out of an organisation. They indicate that almost 60% of young female college nurses express their intent to leave their current posts due to several aspects related to job dissatisfaction (Mudor & Tooksoon 2011:41).

Staff turnover is naturally exacerbated by the fact that losing high performing individuals affects the productivity of an organisation and in hospitals, it affects the provision of quality of care. Staff turnover of skilled professional nurses is viewed as a loss of intellectual capital. Oosthuizen and Ehlers (2007:14) explain that the loss of intellectual capital refers to the depletion of skilled people whose contribution is vitally important for improving the national economy because they are expected to provide a service.

A healthcare organisation that loses skilled professional nurses experiences increased human resource costs and organisational ineffectiveness whilst dedicated professionals are able to increase their salaries in alternative employment and to better support their families (Kingma 2007:1291). According to Swansburg (1996:139), movement of employees from an organisation or health care institution results from resignations, transfers out of the organisational units, discharges, retirement, and death. According to Mathis and Jackson (2003:78), staff turnover is described in a number of different ways, including voluntary versus involuntary turnover, functional and dysfunctional turnover, as well as controllable and uncontrollable turnover. Voluntary turnover is caused by many factors, including improved career opportunities, remuneration, supervision, demographic issues and personal reasons. Involuntary turnover, on the other hand, is triggered by employees who are not complying with organisational polices and work rules, thus not meeting the expected performance standards.

Windle (2008:211) mentions that high nursing staff turnover affects the goal of hospitals to provide quality care for patients. Professional nurses are viewed as important to hospitals because they represent the majority of health professionals and they work 24 hours per day providing care to patients, whilst other health professionals spend relatively little time with the patients. Oosthuizen and Ehlers (2007:22) support this point of view by pointing out that nurses constitute the largest professional group in the South African health services and form the backbone of nursing care. Since professional nurses are a critically important group in the provision of care to patients, it is essential that nursing personnel numbers, including the category of professional nurses, are sufficiently covered for day, evening, and night shifts. With a high staff turnover rate, such a staffing pattern is impossible to achieve because the experienced nurses are spread thinly amongst nursing care units, which complicates the task of providing patient care in South Africa (Hall & Buch 2009:6). In 2008, there was an average of 40.3% vacant professional nursing posts. The Eastern Cape Province had the highest percentage of vacant posts (53.6%), followed by the Free State (51.3%), Limpopo (43.7%), KwaZulu-Natal (39.6%), and Gauteng (34.4%) (Health System Trust 2008).

## Problem statement

There seemed to be a frequent movement of professional nurses to other healthcare institutions which impacted nurse managers negatively, since they had to ensure that care was given despite having a minimal number of nurses.

It was against this background that the study aimed at determining the experiences of nursing unit managers with regard to turnover of professional nurses at Mokopane Hospital in the Waterberg district of the Limpopo Province, South Africa.

## Research methodology

### Design

A qualitative, explorative, descriptive research method was used because it was a holistic, inductive approach interested in getting rich verbal descriptions of the phenomena being studied as it occurred naturally based on direct observations (Brink, Van der Walt & Van Rensburg 2012:4). The researchers were interested in the meaning of the sense the nursing unit managers made of professional nurses’ turnover in the context of this study and how it affected the working situation in the nursing care units of the Mokopane Hospital (Brink et al. 2012:114). The explorative research design was realised in this study through the researchers asking one main question that was followed by clarity-seeking questions during the interviews with the participating nursing unit managers. The descriptive part of this study was achieved by affording the participants an opportunity to describe their experiences related to the problem studied (Babbie & Mouton 2010:270; Brink et al. 2012:115).

### Study site

The study was conducted at the Mokopane Hospital which is situated five kilometres away from the Mahwelereng Township in the Waterberg district of the Limpopo Province, South Africa. The Mokopane Hospital has 13 units, serving 20 nearby villages with a total of 152 nursing staff members and 12 nursing unit managers.

### Population and sampling

The population for this study was all nursing unit managers at the Mokopane Hospital who managed the nursing care units. Non-probability purposive convenience sampling was used in this study to include all nursing unit managers until data saturation was reached during the interviews conducted.

### Data collection method

Semi-structured one-on-one interviews were conducted to explore and describe the experiences of nursing unit managers with regard to the turnover of professional nurses which they were experiencing in their units at the Mokopane Hospital. Any nursing unit manager who was on either day or night duty during the period of data collection participated. The main question asked to all participants was:

‘Could you kindly explain your experiences with regard to the turnover of professional nurses in your institution.’

A voice recorder was used to capture all interview discussions and field notes were written to supplement the voice recordings. Follow-up interviews were also conducted to clarify data collected and to further verify whether the collected data represented what the participants had said.

## Data analysis

Tesch’s open coding technique, as outlined in Botma et al. (2010:223) was used to analyse data in this study.

### Measures to ensure trustworthiness

To ensure trustworthiness throughout the study, credibility and dependability were observed.

Credibility was ensured through prolonged engagement in the study field with the participants during data collection over a period of two months, during which follow-up interviews were conducted in order to seek clarification about data collected and also to verify whether the analysed data captured correctly what the participants had said during the interviews (Babbie & Mouton 2010:277; Brink et al. 2012:172). Data were collected until saturation was reached.

Dependability was ensured by the full description of the research methods used in the study to enhance the possibility of repeating the study by another researcher in another context (Babbie & Mouton 2010:278).

## Ethical considerations

Ethical clearance was obtained from the University of Limpopo, Medunsa Research Ethics Committee (MREC). Permission to conduct the study was obtained from the Limpopo Department of Health and from the Chief Executive Officer (CEO) of Mokopane Hospital. The participants were informed about the purpose of the study, the need for signing a consent form, and voluntary participation, which emphasised that they could withdraw from the study without punishment.

## Discussion of findings

The findings are based on the five categories and their sub-categories that have emerged from data analysis using Tesch’s open coding technique as outlined in Botma et al. (2010:223). The categories are: Experiences related to staff turnover of professional nurses, feelings related to staff turnover, avoidable versus unavoidable contributory factors of staff turnover, effects of staff turnover on an institution, and suggestions for dealing with staff turnover (Table 1).

**TABLE 1:** Categories and sub-categories reflecting experiences of nursing unit managers at the MokopaneHospital.

Categories	Sub-categories
1. Experiences related to staff turnover of professional nurses	1.1 Working overtime problematic. 1.2 Increased conflict amongst nurses. 1.3 Increased absenteeism.
2. Feelings related to staff turnover	2.1 Feelings of frustration experienced. 2.2 Reduced interest to remain in the organisation.
3. Avoidable versus unavoidable contributory factors of staff turnover	3.1 Poor working conditions. 3.2 Lack of support from top management. 3.3 Family related factors.
4. Effects of staff turnover on the institution	4.1 Provision of patient care compromised due to loss of competent personnel. 4.2 Increased complaints from staff members. 4.3 Recruitment and advertisements costs.
5. Suggestions for dealing with staff turnover	5.1 Support by all management levels. 5.2 Implementation of team building activities. 5.3 Implementation of personnel development programmes.

### Category 1: Experiences related to staff turnover of professional nurses

The study revealed that nursing unit managers had several experiences related to staff turnover of professional nurses which included problems professional nurses had with working overtime, increased conflict amongst nurses and increased absenteeism.

### Sub-category 1.1: Problems in relation to working overtime

Most of the nursing unit managers stated that nurses were obliged to work overtime in order to solve staff shortage problems. This was confirmed by a participant who indicated that:

‘professional nurses are always to work overtimes during the days on which the units are not covered well and you will even feel guilty as a manager doing that because you know very well that you always ask this person to work overtime, always. Another issue is that you know that this person that you are asking to work overtime has not rested enough.’

Another participant with the same view indicated that:

‘you [*know*] that these professional nurses who are requested to work overtime, they just agree because maybe they don’t want to resign, but it is a problem because you will find that this person is tired but you keep on asking her.’

Olds and Clarke (2010:153) support the finding as indicated in their report about several adverse effects on professional nurses of extended working hours that seek to solve nursing shortages.

### Sub-category 1.2: Increased conflict amongst nurses

The study revealed that staff turnover of professional nurses caused conflict amongst the remaining professional nurses. Most participants mentioned increased incidents of conflict due to work overload amongst the remaining staff members. This is reflected in the following excerpt:

‘You know, we are experiencing a problem as managers because of this problem of nurses resigning because it causes fights amongst nurses who are supposed to remain at work to provide care to patients. They are always quarrelling about who is supposed to remain at work covering shortage. Sometimes you have this feeling that this is true, but there is no option; they must just work.’

Additionally, another participant said:

‘You know, nurses are fighting always because they no longer want to cover the shortage; when you request, then they will refuse, stating the reasons that it is not long that they worked and they will refer you to another nurses and the other nurse will also tell you how they are fighting about this issue [*of relieving one another*].’

The study conducted by Park (2007:5) stated that nurses become emotionally exhausted and stressed out to such an extent that they even started exhibiting depersonalised behaviour amongst one another.

### Sub-category 1.3: Increased absenteeism due to fatigue

Increased incidents of absenteeism were mentioned by almost all participants. Staff turnover resulted in increased absenteeism in all units; that was evident from the following direct quotation from one participant:

‘I have observed that in all units when taking rounds either on night duty or day duty there is absenteeism of nurses which emanated from staff turnover. Nurses are absenting themselves from work, especially after working overtime and this reflect[*s*] that they just agree to work overtime and due to tiredness then they absent themselves.’

According to Portnoy (2011:49), nurses experience fatigue due to burnout related to several aspects, such as workload increased by colleagues who are absent from work and due to work-related stress of providing care to trauma patients.

### Catergory 2: Feelings related to staff turnover

The results of this study reflect that nursing unit managers are not comfortable with the turnover of professional nurses.

### Sub-category 2.1: Feelings of frustration experienced

The participants observed that the decreasing complement of professional nurses experienced frustrations at multiple levels, including at the level of the unit nursing managers. That was confirmed by the participant who indicated:

‘You know, it is frustrating as a manager to see nurses being overworked whilst you don’t have [*a*] solution to the problem. Professional nurses in the units are also frustrated because they have to make plans of making sure that there is someone who must stay behind providing care and also supervising junior personnel.’

Another participant indicated that:

‘I am frustrated because the institution does advertise posts but the people who are hired turn to be less than those who are leaving and this frustrate[*s*] the whole management team.’

In support of the findings, it is stated that nurses experience burnout that results from frustrations that they experience in the working environment due to shortage of nurses (Erickson & Grové 2008).

### Sub-category 2.2: Reduced interest to remain in the organisation

The findings revealed that the nursing unit managers were no longer interested in working at the hospital due to the fact that most professional nurses were resigning from the hospital, leaving them with the burden of an increased workload. An explanation given was:

‘You know, that it is difficult to manage in an institution that [*sic*] there is a lot of staff turnover and currently I am no longer interested in working here as a manager and I am deciding that if I get a better place I will leave this place.’

Another participant with the same feeling said:

‘I am no longer interested in working here because as a manager you always solve problems related to shortage of staff and as you know, it affects all areas of care, I am telling, I am tired working here as a manager.’

In support of the findings, Dieleman and Harnmeijer (2006:1) pointed out that some of the factors influencing health workers to lose interest in their work are job dissatisfaction, financial considerations, and unbearable working conditions.

### Category 3: Avoidable versus unavoidable contributory factors of staff turnover

The findings reveal that there are avoidable versus unavoidable contributory factors to staff turnover at the Mokopane Hospital, which is reflected in the sub-categories of this theme. The sub-categories are poor working conditions, lack of team work, lack of support from top management, and family-related factors.

### Sub-category 3.1: Poor working conditions

Avoidable factors, such as poor working conditions, were said to be contributing to staff turnover because the professional nurses find them intolerable. One of these factors was outlined by the participant who said:

‘the working conditions in this hospital is [*sic*] the ones that lead to nurses resigning. To mention a few, there are no material resources to execute nursing care, the issue of working a lot of overtime, and colleagues who are not cooperating while they can see that some of this [*sic*] things are difficult to solve. All this are intolerable conditions which people feel that this environment is not conducive for one to work in.’

According to Dieleman and Harnmeijer (2006:1), staff members are sometimes forced to leave the institutions due to poor working conditions that are not responsive to the needs of the patients and the health care providers.

### Sub-category 3.2: Lack of support from top management

The findings reveal that there is lack of support from top management during the execution of nursing activities at the hospital, which was evident from the following quotation:

‘Our top management are the ones who contribute sometimes to this staff turnover. Professional nurses are trying their best to work overtime in order to cover up the shortage of professional nurses, the problems come up when a mistake can occur. They don’t even want to listen to explanations that you give; what they want to do is punish these professional nurses. This I think is one other thing that leads to these nurses to leave the organisation.’

The first principle in nursing leadership is to design a safe system where the health care system can support nurses in their work environment whilst providing care to patients; however, nurse leaders lack that support of policy makers (Emerson 2015:3).

### Sub-category 3.4: Family related factors

Staff turnover is sometimes caused by unavoidable family issues of the professional nurses: a nurse may be obliged to request a transfer in order to live with in-laws or a husband in other areas. This was confirmed by the participants who indicated that:

‘some professional nurses, due to marriage, they are supposed to take transfers and relocate to stay with their new families in other provinces or districts. Another factor is that you find that a professional nurse will indicate that at home she is the one who takes care of the siblings and when the parents pass on she has to go back home and work in the nearby hospital.’

Netswera, Rankhumisi and Mavundla (2005:39) outline that the management of an organisation has to assist its employees to maintain the balance between work and family matters to avoid staff turnover related to family matters.

### Catergory 4: Effects of staff turnover to an institution

There were several effects of staff turnover on the hospital that emerged in the sub-categories in relation to staff turnover: is provision of patients’ care compromised due to loss of competent personnel, decreased productivity during provision of care, increased complaints from staff members, as well as recruitment and advertisement costs.

### Sub-category 4.1: Provision of patients’ care compromised due to loss of competent personnel

The findings emphasise that the provision of patients’ care is compromised due to loss of competent personnel. This was described by the participant who said that:

‘this moving out of professional nurses from this hospital has serious effects on provision of quality care, because nurses work only to finish routine. They are no longer providing expected care and this sometimes lead to patients’ conditions becoming worse. The other thing is that you find people who are leaving are experienced and competent and when we hire new nurses you find that they are newly qualified and they still need mentorship.’

### Sub-category 4.2: Increased complaints from staff members

The findings confirm that there are a lot of complaints from the nurses in the unit that are based on the fact that they are overworked. This was indicated in the following excerpt from an interview with a participant:

‘This days, we receive a lot of complains, especially when taking rounds with doctors in the unit, nurses are bitterly complaining about this shortage of personnel and nurse managers always solve a lot of fighting between professional nurses.’

### Sub-category 4.3: Recruitment and advertisements costs

The nursing unit managers indicated that the staff turnover at the hospital increased the costs related to recruitment and also advertisement. This was confirmed by the participant who indicated that:

‘this hospital is losing a lot of money by advertising posts continuously because the process of advertisement of posts is costly.’

Another participant with the same opinion said:

‘the advertisement of post [*sic*] is done very now and then and advertising posts cost a lot of money meaning the institution is spending a lot of money on recruitment and advertisement of posts.’

### Catergory 5: Suggestions for dealing with staff turnover

The following sub-strategies emerged in this category, which suggest the need for strategies to prevent staff turnover; including support by all management levels, implementation of team building activities, and implementation of personnel development programmes.

### Sub-category 5.1: Support by all management levels

The participants suggested that all management at the hospital has to support all categories of nurses with the view of encouraging them to stay at the institution. This was supported by the following quotation:

‘I suggest that management at all levels, need to support the nurses; especially when mistakes occurs [*sic*] in their line of duty. They need to listen to their explanation and stop harassing them but show support. Another thing is that, even if there is punishment that a nurse has to get, it shouldn’t be that we want this nurse to suffer but to correct a wrong behaviour which occurred.’

Managers and leaders in hospitals have to provide support to employees with the purpose of enabling them to achieve set objectives of the hospital by taking more responsibility whilst executing their work (Effken et al. 2010:190).

### Sub-category 5.2: Implementation of team building activities

It was suggested by the nursing unit managers that the top management must create a platform where team building can be encouraged and initiated. This was suggested by the participant who said:

‘I would suggest that, executive management draw strategic plan they must include budget for team building activities and outings because this might encourage the nurses not to leave the institution as this is also therapeutic.’

Spencer-Laschinger and Fida (2014:26) indicated that authentic leaders need to initiate training programmes for their staff members so that they can cope with their work expectations.

### Sub-category 5.3: Implementation of personnel development programmes

Suggestions from the nursing unit managers for decreasing the staff turnover of professional nurses included the fact that there was a need for management to initiate the staff development programmes, which might be appreciated by staff members and might encourage them to stay at the institution. This was the opinion of a participant who said:

‘I suggest that the top management have to initiate a lot of staff development programmes, such as on-job training and in-service education, especially to the newly employed employees, which might assist in keeping personnel in this hospital.’

Spencer-Laschinger and Fida (2014) state that:

in order to keep employees at an organisation, leaders need to consider creating a supportive work environment through development programmes that are sensitive to the employees’ needs. (p. 22)

## Conclusion and recommendations

Staff turnover issues are seen as a global problem and managers have experienced it on multiple levels, based on the findings of this study. Since they are aware of the contributory factors, their effects on individuals and the institution, these problems have to be addressed. Staff turnover should be addressed through several strategies the top management at the hospital can employ. For example, management could create and maintain a healthy environment with resources that nurses need to use during the provision of care, address the effects of the staff turnover, and top management has to support the staff members instead of intimidating them when problems arise.
